# Understanding Cognition, Oxytocin, and Pain in Elders (UCOPE): protocol for a double-blinded cross-over trial in chronic knee osteoarthritis pain

**DOI:** 10.1186/s13063-024-08715-4

**Published:** 2025-02-07

**Authors:** Yenisel Cruz-Almeida, Soamy Montesino-Goicolea, Pedro Valdes-Hernandez, Zhiguang Huo, Roland Staud, Natalie C. Ebner

**Affiliations:** 1 Department of Community Dentistry and Behavioral Science, University of Florida, Gainesville, USA; 2 Pain Research and Intervention Center of Excellence, University of Florida, Gainesville, USA; 3 Department of Neuroscience, College of Medicine, University of Florida, Gainesville, USA; 4 Center for Cognitive Aging and Memory, McKnight Brain Institute, University of Florida, Gainesville, USA; 5 Claude D. Pepper Older American Independence Center, University of Florida, Gainesville, USA; 6 Department of Biostatistics, College of Public Health & Health Professions and College of Medicine, University of Florida, Gainesville, USA; 7 Department of Rheumatology, College of Medicine, University of Florida, Gainesville, USA; 8 Department of Psychology, College of Liberal Arts and Sciences, University of Florida, Gainesville, USA

**Keywords:** Aging, Disability, Knee osteoarthritis pain, Brain, Oxytocin, Randomized controlled trial

## Abstract

**Background:**

Osteoarthritis (OA) is the leading cause of disability among older adults with the knee being the most affected joint. Specifically, there is an urgent need to develop better analgesics for individuals with OA-related pain, since currently used analgesics frequently fail to provide adequate relief or must be discontinued owing to adverse effects. A promising treatment is the neuropeptide oxytocin (OT), which has been shown to play a role in endogenous analgesia with human and animal studies demonstrating anti-nociceptive effects. The primary aims of the study are to examine preliminary analgesic effects of a chronic OT intervention in community-dwelling older individuals with chronic knee osteoarthritis pain.

**Methods:**

In this article, we describe the rationale and design of the Understanding Cognition, Oxytocin, and Pain in Elders (UCOPE) study, a double-blinded intervention in which 80 participants over 45 years of age with knee osteoarthritis pain will be recruited to participate in a cross-over trial of 4 weeks of intranasal oxytocin or placebo administration. Primary study outcomes include preliminary changes in pain intensity and interference as well as multi-modal assessment batteries including circulating biomarkers and neuroimaging measures. Self-reported and quantitative outcomes will be assessed at baseline, post-intervention periods, and up to a 6-month follow-up period.

**Discussion:**

This study will establish preliminary effectiveness of a novel intervention in middle to older aged adults with knee osteoarthritis pain. Achievement of these aims will provide a rich platform for future intervention research targeting improvements in pain and disability among geriatric populations and will serve as a foundation for a fully powered trial to examine treatment efficacy and potential mechanisms of the proposed intervention.

**Trial registration:**

ClinicalTrials.gov NCT03878589. Registered on March 18th, 2019.

## Introduction

### Background and rationale {6a}

Osteoarthritis (OA) is the leading cause of disability among older adults [1, 2] with the knee being the most affected joint [3]. Lifetime risk of developing symptomatic knee OA exceeds 40%, and OA prevalence in the U.S. population is rising due to increased age and obesity [4]. With the number of adults aged over 65 years expected to double from 40 to 88 million by the year 2050, health concerns related to OA for older individuals and society at large will increase in the coming decades. This demographic encompasses a large and growing group in need of better therapeutic modalities that are based on mechanistic translational research. There is an urgent need to develop better analgesics for individuals with OA-related pain, since currently used analgesics frequently fail to provide adequate relief or have to be discontinued owing to adverse effects [5]. 

Historically, OA has been conceptualized as a regional pain condition with symptoms driven by peripheral pathophysiology. However, a growing body of evidence supports the notion that both local and low-grade systemic inflammation contribute not only to articular damage in OA, but also to pain and reduced physical function [6–9]. The poor correspondence between measures of disease severity and clinical symptoms suggests that factors above and beyond peripheral tissue damage must contribute to OA-related pain [10]. Indeed, recent studies demonstrate widespread abnormalities in brain structure, function, and biochemistry in persons with symptomatic OA [11–14], including greater levels of metabolites associated with brain inflammation [15]. Findings from quantitative sensory testing (QST) also demonstrate a maladaptive pain-modulatory profile in symptomatic knee OA [16–18], characterized by heightened pain facilitation and impaired pain inhibition, consistent with significant changes in pain processing at multiple levels of the neural axis (i.e., brain and spinal cord). Furthermore, individuals with severe knee OA pain experience significant maladaptive psychological characteristics consistent with changes in brain regions associated with mood and emotional function [11, 19–21].

A promising treatment candidate for pain that is increasingly discussed in the literature, is the neuropeptide oxytocin (OT) [22–24]. While best known for its roles in parturition and lactation [25], OT has been shown to play a role in endogenous analgesia with animal studies demonstrating anti-nociceptive effects [26–28]. In humans, low plasma OT levels are associated with an increased prevalence of chronic pain [29–31], and acute (i.e., one-time) intranasal OT administration decreases experimental pain sensitivity, increases pain inhibition, and improves mood and positive affect in younger individuals [32, 33]. However, the analgesic effects of chronic OT administration remain understudied in persons with chronic pain, and in middle-to-older aged adults affected by symptomatic knee OA. The proposed work will address this research gap.

**Fig. 1:**
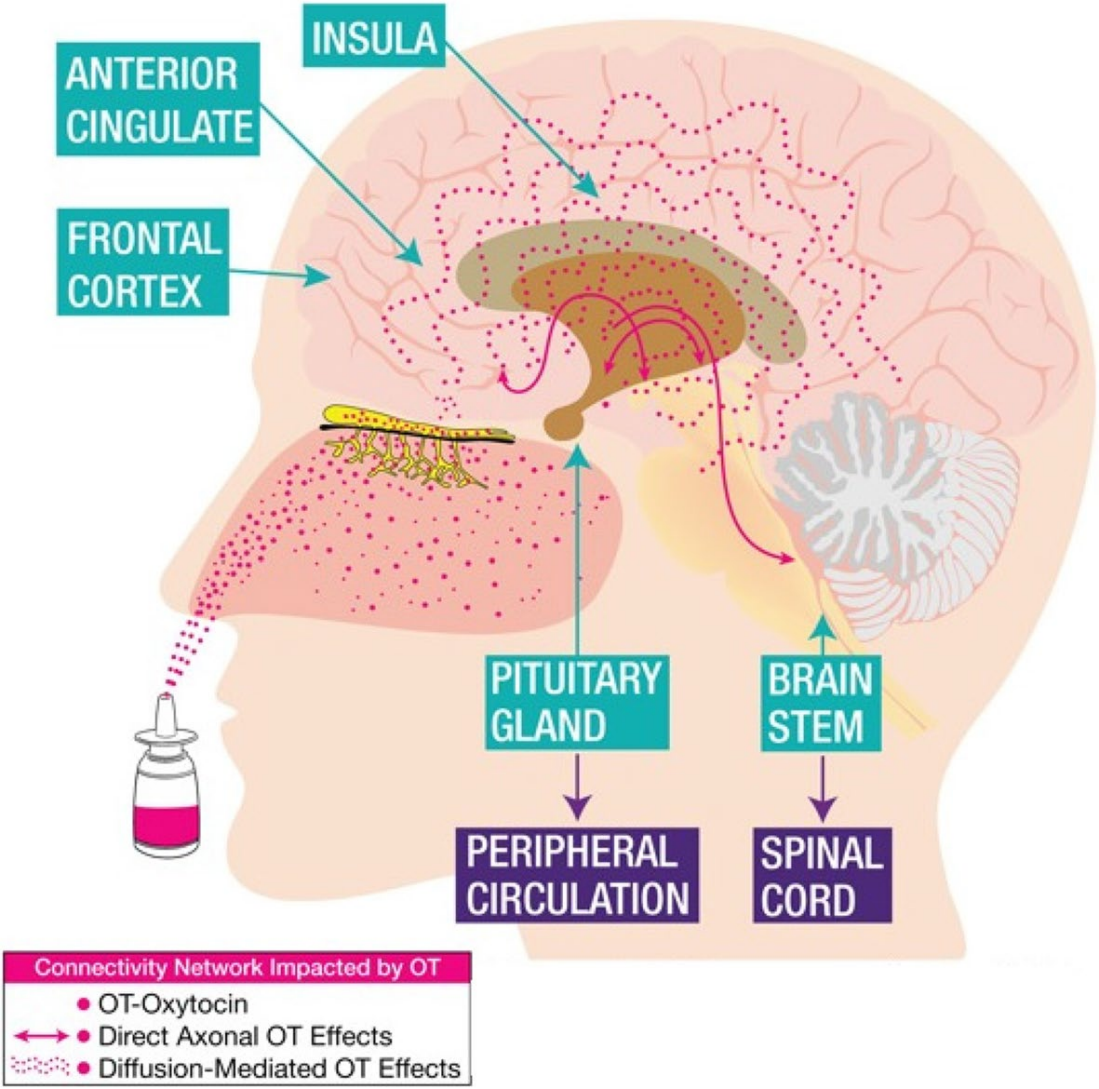
Widespread effects of OT in the brain, spinal cord, and peripheral circulation

A notable characteristic of OT is that it is multi-functional in ways that may be leveraged therapeutically [22]. OT’s potential analgesic mechanisms may be explained by its roles as both a neurotransmitter and a paracrine hormone. Figure 1 shows the widespread effects of OT in the brain, spinal cord, and peripheral circulation. As a neurotransmitter, OT may provide analgesia via widespread effects on the brain [34, 35] and spinal cord [34]. As a hormone, OT is released into the peripheral circulation and acts directly on multiple organ systems. Possibly connecting these two routes, the OT-secreting system within the hypothalamus integrates neural, endocrine, and immune function [35]. Indeed, animal studies suggest that OT inhibits inflammation both in the brain and spinal cord [34], with recent evidence supporting that OT’s effect on inflammation contributes to its analgesia. Similarly, in humans, OT decreases pro-inflammatory cytokines in the peripheral circulation [36]. Further, OT’s effects in mood and affect regulation are likely explained by its interaction with the hypothalamic–pituitary–adrenal (HPA) axis and effects on stress regulation [35, 37, 38].

### Objectives {7}

Based on the combined evidence presented above, and on our working hypothesis that middle-to-older adults with chronic knee OA may benefit therapeutically from OT’s multiple pain-modulatory mechanisms, the objective of the Understanding Cognition, Oxytocin, and Pain in Elders (UCOPE) study is to determine the analgesic effects of a chronic OT intervention in community-dwelling middle-to-older individuals with chronic pain.

### Trial design {8}

This is a proof-of-principle mechanistic cross-over, randomized, double-blinded trial designed to examine the preliminary analgesic effects of a 4-week intranasal oxytocin administration and the extent to which positively affects psychophysical function as well as brain and circulating markers of inflammation with a 1-to-1 allocation ratio.

## Methods: participants, interventions, and outcomes

### Study setting {9}

This trial is conducted at the University of Florida (UF) Health Sciences Center, Dental Tower and McKnight Brain Institute, approved by the University of Florida IRB (IRB#201,801,467, Protocol IRB version 10, IRB approved on 11/5/2020), and registered at ClinicalTrials.gov (NCT03878589). The UCOPE Study adopts multiple strategies to promote open and consistent communication within the study team, including (1) biweekly videoconferences attended by investigators and study staff, (2) online discussion groups to facilitate communication regarding protocol issues or study concerns using the Microsoft TEAMS platform, and (3) investigator meetings for intensive review and discussion of the protocol, amendments, and for recalibration of study staff, as needed. These communication strategies are designed to provide timely responses to questions or concerns that arise over the course of the study and are taking place as remote activities as needed (e.g., during the COVID-19 pandemic).

### Eligibility criteria {10}

The UCOPE Study aims to enroll 80 adults over 45 years of age who meet the American College of Rheumatology (ACR) Clinical Criteria for knee OA. Our inclusion and exclusion criteria are designed to facilitate enrollment of a representative sample of adults with knee OA while optimizing participant safety and rigor of study findings.

#### Inclusion criteria

Participants over 45 years of age with unilateral or bilateral symptomatic OA of the knee based on the 1986 American College of Rheumatology (ACR) criteria [39], which are:

- Knee pain, plus at least 3 of the following 6 signs/symptoms:
- Age > 50 years, -month
- Morning stiffness < 30 min,
- Crepitus,
- Bony tenderness,
- Bony enlargement, and
- No palpable warmth.

Individuals with knee OA who also report pain in other sites of the body are eligible, as long as the knee is the primary site of pain. People of both sexes and various ethnic/racial backgrounds are recruited, and it is anticipated that the sample will include approximately 65% female and 35% male, based on the prevalence of symptomatic knee OA.

#### Exclusion criteria

Participant exclusion criteria are based on OT, pain, and/or MRI criteria as well as any information that may confound study measures and has been detailed in Table 1.

**Table 1:** Inclusion and exclusion criteria for the *UCOPE* study participants

• **OT application** o Hypersensitivity to OT or vasopressin o History of hyponatremia o Syndrome of inappropriate antidiuretic hormone secretion, or psychogenic polydipsia o On vasoconstrictors such as desmopressin, pseudoephedrine, or antidiuretic medication, or anti-inflammatory drugs, or muscle relaxants o Low sodium and high osmolality levels o Blurred vision caused by a medical condition o Excessive smoking or excessive drinking o Muscle pain because of systemic disease o Significant nasal pathology o Previous or concurrent use of narcotics delivered intranasally (e.g., cocaine), or gastroparesis o Individuals with heart problems (e.g., cardiomyopathy, history of myocardial infarction, arrhythmias, prolonged QT interval) o Pregnancy or breastfeeding
• **Pain** o Concurrent medical or arthritic conditions that could confound symptomatic knee OA-related outcomes or coexisting disease that could preclude successful completion of the protocol including systemic rheumatic condition (e.g., rheumatoid arthritis, systemic lupus erythematosus, fibromyalgia) o History of clinically significant surgery to the index knee o Uncontrolled hypertension (> 150/95) o Poorly controlled diabetes (HbA1c > 7%) o Neurological disease (e.g., Parkinson’s, Multiple Sclerosis) o Cardiovascular or peripheral arterial disease o Serious psychiatric disorder requiring hospitalization within the past twelve months or characterized by active suicidal ideation o Diminished cognitive function that would interfere with completion of study procedures (i.e., Montreal Cognitive Assessment (MoCA) score < 25) o Ongoing participation in another research study
• **Magnetic resonance imaging** (note that participants can participate in the UCOPE Study but not in the MRI/MRS portion of the study) o Large pieces of metal in the body or metal in the face or neck o Claustrophobia o Major medical surgery in the past two months o History of brain surgery or any serious brain condition like aneurysm, stroke, or seizures

### Who will take informed consent? {26a}

Potential participants complete a scripted phone screening to determine initial eligibility. These phone interviews are conducted by trained study staff using standardized scripts. The interview is divided into three parts: (1) initial explanation of the study and obtaining verbal consent to proceed with the interview; (2) collection of contact information and demographic data; and (3) screening interview to determine eligibility via phone. If eligible over the phone, participants are scheduled for the first study visit, at which time verbal as well as written informed consent is obtained by the study research coordinator and final eligibility determined. Personal information about potential and enrolled participants will be collected, shared, and maintained using unique participant IDs in order to protect confidentiality before and during the trial. After the trial, all 18 PHI identifiers will be removed to completely anonymize the data.

### Additional consent provisions for collection and use of participant data and biological specimens {26b}

Participants also consent of collection and use of their data including biological specimens in future research.

### Interventions

#### Explanation for the choice of comparators {6b}

We chose an intranasal placebo nasal spray that looks identical to the oxytocin nasal spray bottle and contains the exact same ingredients as the oxytocin spray, but without the oxytocin.

### Intervention description {11a}

#### Intervention

The UCOPE Study is a double-blinded, cross-over design that randomizes middle to older age adults (age over 45 years) with symptomatic knee OA pain to 4 weeks of intranasal self-administration of either OT or P (48 IUs daily) followed by a 4-week washout period and a second 4-week intervention (either OT or P). Individuals who consent to participate in the study and are deemed eligible after the initial screening undergo three baseline sessions (health, sensory, and neuroimaging) for collection of clinical, functional (physical, cognitive, emotional), QST, and brain imaging, with each session lasting 2–3 h. On the last baseline session, participants self-administer the nasal spray assigned to them for the arm. There is a 35-min delay to allow for effects to occur, and then a shortened version of the QST is performed. This will allow us to compare the acute effects of the spray with chronic administration. During the intervention, participants then self-administer twice daily 24 IUs intranasal OT or P (once in the AM and once in the PM for a total of 48 IUs daily) following standardized intranasal OT administration guidelines [40] and are contacted by phone once a week for assessment of adverse effects. Participants are also asked to wear devices during their intervention that measures their activity and health data; and they complete a diary on two weekdays and one weekend day during each of the intervention weeks comprising measures of pain, mood, social engagement, and stress.

During the last week of each intervention period (day 21 to 28 of their intervention), participants attend three assessment sessions (referred to as post-intervention sessions) that are identical to the baseline sessions. On the mornings of the post-intervention assessment days, participants do not apply the OT/P nasal spray, to avoid confounding of acute and chronic effects.

After the post-intervention visits, individuals have a 4-week washout period during which they do not perform any study-related activities. After this period, participants repeat the baseline, intervention, and post-intervention phases, but instead receive the alternative treatment they did not receive in trial phase one. Study participation is expected to occur over a duration of 3–5 months. After completion of both arms, participants are contacted after 1 week, 3 months, and 6 months via phone interview for a follow-up health checkup.

#### Intervention: intranasal oxytocin (OT) or placebo (P)

During the 4-week intervention, participants self-administer via intranasal spray 24 IUs of OT or P twice a day at home, at 7–9AM and again at 5–7PM. Compliance is monitored by using a log to fill in each day during the intervention and measuring the fluid left in the spray bottle after the treatment period. If at any time during the treatment it is determined that the participant should not continue due to adverse events, the participant is discontinued. Furthermore, at baseline and at the end of the intervention, blood and urine samples are checked (e.g., for osmolality levels, sodium levels, and any other blood markers out of range) to ensure that no adverse changes have occurred during the study intervention. Additional blood is collected for future analysis of circulating levels of oxytocin before and after each intervention phase. Participants are contacted once a week by phone to assess any side effects. These weekly calls also ensure regimen compliance. Importantly, at the post-intervention sessions, we assess changes in health status and participants’ global impression of change (PGIC/MPIC) since starting the intervention.

#### Wash-out period

Participants undergo a 4-week wash-out period in between trial phases one and two. The wash-out period serves to prevent any carryover effect (i.e., the effect of OT/P in phase one will persist in phase two). At the end of the washout, participants begin phase two of the trial, which is identical to phase one except that participants receive the alternate treatment during the intervention (i.e., either OT or P).

### Criteria for discontinuing or modifying allocated interventions {11b}

If at any time during the treatment it is determined that the participant should not continue due to adverse events, the participant is discontinued. Furthermore, at baseline and at the end of the intervention, blood and urine samples are checked (e.g., for osmolality levels, sodium levels, and any other blood markers out of range) to ensure that no adverse changes have occurred during the study intervention. Additional blood is collected for future analysis of circulating levels of oxytocin before and after each intervention phase. Participants are contacted once a week by phone to assess any side effects.

### Strategies to improve adherence to interventions {11c}

Compliance is monitored by using a log to fill in each day during the intervention and measuring the fluid left in the spray bottle after the treatment period. We also use weekly calls to ensure regimen compliance.

### Relevant concomitant care permitted or prohibited during the trial {11d}

Usual care for chronic conditions is allowed and participants are asked to not change any of their ongoing treatments unless medically necessary.

### Provisions for post-trial care {30}

We have no provisions for ancillary and post-trial care and the university has limited compensation for those that may suffer harm from trial participation.

### Outcomes {12}

#### Primary outcome variables

This is a proof-of-principle mechanistic trial has two categories of primary outcome variables: (1) clinical and experimental pain, and (2) mechanistic brain measures. These variables are expected to reflect the complex, multidimensional construct of pain in a clinical population.

##### Clinical and experimental pain

**Fig. 2:**
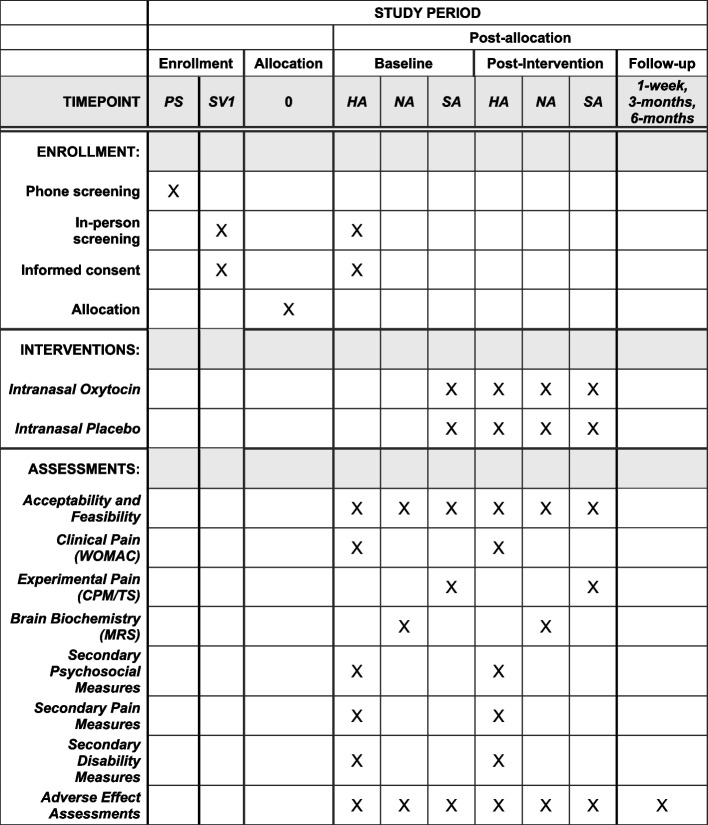
SPIRIT schedule including the schedule of enrollment, interventions, and assessments. HA, Health Assessment Session; NA, Neuroimaging Assessment Session; SA, Sensory Assessment Session; PS, Phone Screening; SV1, Screening Visit 1

A. The primary clinical pain outcome is the Western Ontario McMaster Universities Osteoarthritis Index (WOMAC). The WOMAC [41] consists of 24 items assessing lower extremity symptoms over the past 48 h. Respondents report the severity of each symptom on a 5-point scale where higher scores reflect greater symptom severity. The WOMAC yields three subscales: (1) pain during activities (5 items), (2) daytime stiffness (2 items), and (3) impairments in physical function (17 items). The WOMAC is widely used in knee OA research, including clinical trials, and has shown adequate construct validity and reliability [41, 42]. The WOMAC will be assessed during the Health Assessment Sessions (HA in Fig. 2).

B. The primary experimental pain outcome measure is a composite score that combines conditioned pain modulation (i.e., pain inhibition) with temporal summation (i.e., pain facilitation). Conditioned pain modulation (CPM) is conducted as in our previous work [43–46]. Briefly, heat is applied to the midline portion of the left forearm increasing at a rate of 1 °C/s, and is discontinued by the participant at pain-40 (pain level of 40/100). The temperature required to produce pain-40 is recorded. The conditioning stimulus is cold-water immersion of the contralateral hand at approximately 10 degrees for 1 min. The test stimulus is then presented immediately after the conditioning stimulus. A pain inhibition score is calculated as first minus last temperature divided by first temperature (× 100), whereby inhibition is denoted by a negative value and pain facilitation by a positive value as recommended by expert consensus [47].

Temporal summation is measured using thermal and mechanical stimuli. Temporal summation of thermal pain is assessed in the medial portion of the ipsilateral forearm of the index knee by verbally rating the intensity of pain evoked by each of five brief, repetitive, suprathreshold heat pulses on a scale of 0 (no pain sensation) to 100 (the most intense pain sensation imaginable). Three target temperatures (44, 46, and 48 °C) are delivered for less than 1 s, with an approximately 2.5-s inter-pulse interval during which the temperature of the contactor returns to baseline (32 °C). The procedure is terminated if the participant rates the thermal pain at 100. The average rating over the 5 trials, an index of overall sensitivity to suprathreshold heat pain, and the maximum increase in pain, a measure of temporal summation, is determined for each participant and used in the analyses later. The latter is calculated by subtracting the first trial rating from the maximum rating provided at each target temperature. For mechanical temporal summation, a nylon monofilament (Touchtest Sensory Evaluator 6.65) that is calibrated to bend at 300 g of pressure is applied to the index knee. First, participants rate the pain intensity experienced during a single application of the monofilament. Then, they rate the maximum pain intensity experienced during a series of 10 contacts applied at a rate of one contact per second. Temporal summation is computed by subtracting the rating of the single stimulus from the rating of the series of 10 stimuli. When creating the composite pain modulatory balance score, the temporal summation component is reverse scored, such that higher composite scores reflect better pain modulatory balance. Experimental pain will be assessed during the Sensory Assessment Sessions (SA in Fig. 2).

##### Brain imaging measures

Our primary brain imaging outcome measure are changes in response to oxytocin administration of multiple prefrontal metabolites reflective of neuroinflammatory processes. MRI images will be acquired with a 3 T Siemens MAGNETOM Prisma (AG, Erlangen, Germany) Scanner (software version VE11C) at the University of Florida’s McKnight Brain Institute. Proton MRS (1H-MRS) will be acquired from a 3 × 3 × 3 cm3 prefrontal brain voxel using a Mescher-Garwood point-resolved spectroscopy (MEGA-PRESS) approach with and without water suppression pulses [2000 ms, 68 ms (TR, TE)]. The prefrontal voxel will be placed medial on the axial plane, aligned with the genu of the corpus callosum, inclusive of genual ACC and medial prefrontal cortex. We will use the LCModel software to quantify the following metabolites: N–acetyl aspartate (NAA), Myo-Inositol (Ins), combined glutamate/glutamine (Glx), total choline (tCho), and total creatine (tCr). These metabolites were chosen because they can be reliably measured, have previously been associated with aging and/or chronic pain, and they provide information about neuronal density and integrity (NAA), glial activation and neuroinflammation (Ins), turnover of cell membrane (tCho), and energy metabolism (tCr). We will also quantify the cerebrospinal fluid fraction composition of the voxel to control in the statistical analysis. During the entire session, pulse and respiration are continually measured using the Siemens scanner physio devices (pulse oximeter and respiration strap). The entire session is no longer than 3 h, of which the participant is in the scanner for no longer than 90 min. Brain measures will be collected during the Neuroimaging Assessment Sessions (NA in Fig. 2).

##### Secondary outcome measures

Additional outcome measures, mediators, moderators, and covariates for the UCOPE Study will be evaluated guided by recommendations by the IMMPACT group for clinical pain trials [48–51]. For example, we will examine treatment effects on several secondary outcome measures, including cognitive and physical function as well as psychosocial factors. To examine the duration of therapeutic responses, we also collect monthly measures of pain intensity for 3 months following the intervention. Additionally, before and after the intervention periods, participants fill out the Goal Attainment Scale as an individualized outcome measure involving goal selection and goal scaling that is standardized to calculate the extent to which a participant’s own goals are met [52]. Secondary outcome measures will be collected during the Health Assessment Sessions (HA in Fig. 2).

##### Psychosocial and pain measures

Multiple psychosocial factors have been related to chronic pain. We assess psychosocial factors across the following broad domains: (1) Pain coping: The Coping Strategies Questionnaire-Revised (CSQ-R) consists of 27 items relating to how individuals cope with pain [53]. (2) Affective distress: The Beck Depression Inventory, 2nd Edition is a widely used depression scale that assesses affective (e.g., sadness, loss of interest), cognitive (e.g., worthlessness, guilty feelings), and somatic (e.g., changes in sleep, tiredness or fatigue) symptoms of depression [54]. The Positive and Negative Affect Scale (PANAS) is a 20-item scale that assesses positive and negative affect [55]. For this study, participants will be requested to provide “state” information by responding to items regarding “the present moment.” (3) Satisfaction/quality of life: We assess self-reported quality of life using the 36-Item SF Survey Quality of Life [56], and the Satisfaction with Life Scale (SWLS) [57] assesses satisfaction with one’s lives as a whole. (4) Sleep: Self-reported sleep quality during the past month is measured using the Pittsburgh Sleep Quality Index (PSQI) [58]. (5) Interoceptive awareness: We assess self-reported interoceptive awareness using the Multidimensional Assessment of Interoceptive Awareness (MAIA) [59]. (6) Chronic stress: Using the Daily Stress Inventory, we collect daily self-reports of sources and individualized impact of relatively minor stressful events [60, 61]. (7) Perceived stress: We will employ the Perceived Stress Scale as a self-report assessment of their perceived stress levels during the last month [62]. (8) Impulsivity: We assess self-reported behavioral inhibition using the Behavioral Inhibition System and Behavioral Activation System (BIS/BAS) scales [63]. (9) Neuropathic pain: Individuals respond to the PainDetect questionnaire to assess neuropathic pain detection. If an individual scores greater than 12, their clinical pain for each area of testing is assessed during the QST session [64]. (10) Empathy: We assess empathy using the Affective and Cognitive Measure of Empathy (ACME) questionnaire [65]. (11) Back pain: If an individual indicates they experience back pain, we assess such pain using the Chronic Low-Back Pain (Minimal Dataset) [66, 67], Oswestry Low Back Pain Disability Questionnaire [68, 69], and Brief Pain Inventory questionnaires [70]. (12) Childhood trauma: Participants will complete the Childhood trauma questionnaire-short form (CTQ-SF) as a retrospective screening tool for childhood maltreatment in adults and the 10-item Adverse and Traumatic Experiences Scale to measure childhood trauma. (13) Resilience: The Brief Resilient Coping Scale captures tendencies to cope with stress adaptively and focuses on the tendency to effectively use coping strategies in flexible, committed ways to actively solve problems despite stressful circumstances. (14) Anger: The State-trait Anger Expression Inventory-2 (STAXI-2) measures the intensity of anger as an emotional state (State Anger) and the disposition to experience angry feelings as a personality trait (Trait Anger). (15) Agitation: The Cohen-Mansgield Agitation Inventory (CMAI) scale will be used to systematically assess agitation.

##### Disability and follow-up measures

The WOMAC Physical Function Scale [41] is used to assess changes in self-reported disability following the intervention. We also use the Short Physical Performance Battery (SPPB) as a measure of lower extremity mobility and function, comprised of three different components: standing balance tasks (side-by-side, semi-tandem, and tandem stance), a 4-m walking speed task, and a repeated chair stand task [71]. The SPPB has been extensively validated and used for assessing lower extremity function among middle-aged and older adults [71, 72], including those with knee OA [73]. Finally, follow-up measures of pain intensity and interference will be collected using the items from the Graded Chronic Pain Scale [74], administered during the phone follow-ups 1 week, 3 months, and 6 months after study completion.

### Participant timeline {13}

A schematic diagram (SPIRIT schedule) is presented in Fig. 2 including the schedule of enrollment, interventions, and assessments.

### Sample size {14}

Based on our pilot study estimates, we plan to recruit 80 participants. Assuming an attrition rate of 20% at the follow-up visits, we will have 64 participants undergo the OT and the P intervention arm at the 4-week post-intervention follow-up visits. This sample size yields a power over 0.80, even based on a very conservative Bonferroni correction for multiple comparisons (i.e., testing twelve hypotheses), which lowers the alpha level from *α* = 0.05 to *α* = 0.004. This power calculation is based on a single-phase design, and the actual crossover design (two phases) has higher statistical power because participants receive both OT and P at different phases and serve as their own matched control.

### Recruitment {15}

The UCOPE Study implements a multimodal recruitment plan that includes community-based and clinic-based strategies. Recruitment methods are developed and continually monitored and modified with support from the UF Clinical Translational Science Institute (CTSI). Community-based recruitment efforts include: recruitment flyers, newspaper ads, radio ads, and ads in other electronic and print media. Additional recruitment strategies include advertisement on social media, direct mailing of recruitment materials, and word of mouth. The CTSI has created an electronic data repository, which allows investigators to query electronic health records to facilitate recruitment of participants. In addition, the UCOPE Study investigative team has relationships with primary care and Rheumatology clinics that see large numbers of patients with knee OA, and recruitment from these clinics will be implemented as needed. Recruitment efforts are titrated to maintain consistent participant flow and avoid excessive wait times. Study recruitment was originally planned to be completed by summer of 2024. However, unforeseen ethical and regulatory changes to study screening and enrollment procedures resulting from one serious cardiac adverse event in February 2024 halted our enrollment. Although we continued to recruit and waitlist for potential enrollment, once we were cleared to continue study enrollment, there was not enough time for new participants to complete the critical safety follow-up before the end of funding in early 2025.

### Assignment of interventions: allocation

#### Sequence generation {16a}

For eligible participants, randomization occurs immediately prior to the first intervention visit. Stratified block randomization is employed, in which participants are randomized to receiving the OT prior to the P treatment (vs. the reversed treatment order) using a 1:1 allocation scheme, stratified by age, sex, education, and MRI eligibility. This randomization approach forms strata (different combinations of age (high/low), sex (M/F), education (high/low), and MRI (Y/N)) ensuring a balanced OT vs. P assignment over time.

This stratified block randomization scheme (phase one of the trial) and a randomization generation formula have been implemented in an Excel spreadsheet. This spreadsheet is accessed by the Investigational Drug Service (IDS) pharmacist who independently enters age, sex, education, and MRI eligibility of a specific participant. The spreadsheet automatically generates the randomization assignment, and the study drug is dispensed accordingly by the pharmacist for phase one of the trial. Since this is a cross-over design, the treatment switches over in phase two of the trial. To be specific, after a wash-out period of 4 weeks, the participant receives the other treatment that they did not receive in phase one.

### Concealment mechanism {16b}

Allocation sequence is concealed to all study investigators and staff via the Excel spreadsheet that is only accessible to the IDS pharmacist.

### Implementation {16c}

Allocation sequence is generated automatically by the spreadsheet after age, sex, education, and MRI eligibility is entered by the IDS pharmacist.

## Assignment of interventions: blinding

### Who will be blinded {17a}

Trial participants, care providers, outcome assessors, data analysts, and all investigative staff except the IDS pharmacist are blinded. As the pharmacist operates independently of the rest of the study team, the double-blind nature of this clinical trial is assured.

### Procedure for unblinding if needed {17b}

As noted above, all study staff and participants remain blinded, and only the study pharmacist, who is not involved in collection of pre- or post-treatment outcome measures, is unblinded regarding the treatment condition. To assist with expectation management, all study visits and outcome measures are delivered to each participant via reading scripts. To determine whether blinding was adequately maintained, participants are asked following the intervention which treatments they believe were received after study completion. Unblinding is only permissible under a necessary medical emergency for the safety and treatment of a trial participant via documented communication between the PIs, and pharmacists alerting the study statistician to ensure that data is recorded.

### Data collection and management

#### Plans for assessment and collection of outcomes {18a}

Assessments and collection of outcomes will be performed at baseline and post-intervention periods during both study phases 1 and 2. Checklists will be employed during each visit to ensure completion of outcomes. Assessors will be (re)trained at least once a year and multiple measurements will be collected to promote data quality. Data collection forms are found in the UCOPE study UF TEAMS site.

### Plans to promote participant retention and complete follow-up {18b}

Plans to promote participant retention will include frequent contact with study team including weekly calls for symptom checks, and reminder calls prior to scheduled study visits. We will aim to collect as much outcome data as possible in participants who discontinue or deviate from the protocol, including detailed records of reasons for discontinuation.

### Data management {19}

A REDCap online data management system is used for the UCOPE Study. This secure system allows online administration of questionnaires, including automation of study-related emails to study participants. The system includes range limits to enhance accuracy of data entry by participants and study staff. The system generates reports for monitoring of enrollment, retention, and other study metrics. The REDCap system is maintained by the UF Clinical and Translational Science Institute (CTSI). To further promote data quality, research coordinators are trained and retrained three times a year. Data management procedures are found in the UCOPE study UF TEAMS site.

### Confidentiality {27}

Personal information about potential and enrolled participants will be collected, shared, and maintained using unique participant IDs in order to protect confidentiality before and during the trial. After the trial, all 18 PHI identifiers will be removed to completely anonymize the data.

### Plans for collection, laboratory evaluation, and storage of biological specimens for genetic or molecular analysis in this trial/future use {33}

UCOPE will collect serum and plasma to evaluate circulating oxytocin and cytokine levels before and after each intervention phase. Serum and plasma will be processed and stored at − 80 degrees until study completion. Whole blood and PAXgene tubes are also collected and stored at − 80 degrees for future ancillary studies.

### Statistical methods

#### Statistical methods for primary and secondary outcomes {20a}

Prior to data analysis, summary statistics will be calculated for demographic characteristics and all predictor and outcome measures at each of the study phases. The intention-to-treat principle will be adopted, in which all randomized participants will be analyzed according to their assigned group regardless of protocol derivation. We use self-reported pain intensity (clinical pain)—one of our primary outcome variables, to demonstrate the statistical modeling procedure, which can be extended to the other primary (experimental pain, brain imaging) and secondary (standardized, multimodal experimental pain batteries, other MRI, and physical, cognitive, and emotional functioning measures) outcomes. To correct for multiple testing, the false discovery rate (FDR) will be calculated by employing the Benjamini–Hochberg method [75]. Below we describe the data analysis plan for each of our aims.

#### Specific Aim 1: determine effects of intranasal OT intervention on clinical and experimental pain

In the crossover design, half of the participants will receive 4-week OT in phase one, followed by 4-week washout period, and 4-week P in phase two, which is denoted as the OP sequence. Similarly, the other half of the participants will undergo the PO sequence. Since the same participant will receive both OT and place in different phases, to consider the correlation of both phases of the same participant, we will fit the following linear mixed model using R package *lmer*:

$${y}_{it}=\mu +{\tau }_{O}I\left({g}_{it}=O\right)+{\tau }_{P}I\left({g}_{it}=P\right)+{\pi }_{t}+{\theta }_{OP}I\left({g}_{i1}=O,t=2\right)+{\theta }_{PO}I\left({g}_{i1}=P,t=2\right)+{\sum }_{j=1}^{J}{\beta }_{j}{x}_{ij} +{\alpha }_{i}+{\varepsilon }_{it}$$

${y}_{it}$ is the change in self-reported pain intensity (before and after intervention) for participant $i$ at phase $t (t=\text{1,2})$; μ is the intercept term; g_it_ is the intervention group for participant $i$ at phase $t$; ${g}_{it}=O$ denotes the OT group, and ${g}_{it}=P$ denotes the P group; $I(\cdot )$ is an indicator function, which equals to 1 if the expression inside (⋅) is true, and 0 otherwise; ${\tau }_{O}$ and ${\tau }_{P}$ are the OT treatment effect and P effect respectively; ${\pi }_{t}$ is the time effect at phase $t$; ${\theta }_{OP}$ is the carryover effect in the OP sequence; ${\theta }_{PO}$ is the carryover effect in the PO sequence; ${x}_{ij}$ is the ${j}^{th}$ covariate for participant $i$ and the covariates include baseline outcome variables, age, sex, race, etc. ${\beta }_{j}$ is the coefficient for the ${j}^{th}$ covariate; ${\alpha }_{i}\sim N\left(0,{\sigma }_{\alpha }^{2}\right)$ is the subject-specific random effect; ${\varepsilon }_{it}\sim N\left(0,{\sigma }^{2}\right)$ is the random noise term.

#### Specific Aim 2: characterize the neurobiological mechanisms underlying interindividual differences in analgesic response to intranasal OT intervention

To test this hypothesis, we will perform mediation analysis, where the change in pain intensity is the outcome variable, the intervention is the predictor, and brain metabolites are the mediators, adjusting for sex, age, race/ethnicity, BMI, medication use and accounting for changes in joint pathology, and other relevant factors and confounders. The mediation effects and their 95% confidence interval will be determined using the R mediation package.

### Interim analyses {21b}

This is a proof-of-principle mechanistic trial that is not powered for any interim analysis. The DSMB will make the recommendation of stopping the trial if there is a change in the risk-to-benefit ratio.

### Methods for additional analyses (e.g. subgroup analyses) {20b}

Though our study implements a 4-week washout period to minimize carryover effects, we will perform statistical tests to formally determine the impact of potential carryover effects. Statistically speaking, instead of examining no carryover effect, a relaxed criterion is to examine whether the carryover effects in the OP sequence and the PO sequence are the same (i.e., ${\theta }_{OP}={\theta }_{PO}$), which is a sufficient condition to estimate the OT effect in a crossover design. If there is no significant difference between ${\theta }_{OP}$ and ${\theta }_{PO}$, we will proceed to examine the treatment effect by taking advantage of the crossover design (See Sections below). If there is a significant difference between ${\theta }_{OP}$ and ${\theta }_{PO}$, we can only use data from phase one to estimate the OT effect (See Sections below).

For estimation of the OT effect under the scenario that the carryover effect has no impact, we will be able to estimate the OT treatment effect in comparison to the *P* effect (i.e., ${\tau }_{O}-{\tau }_{P}$). We will fit the above linear mixed models, and obtain estimate, 95% CI, and *p* value for ${\tau }_{O}-{\tau }_{P}$.

For estimation of the OT effect under the scenario that the carryover effect has impact, we cannot estimate the OT treatment effect from the above linear mixed model. We will have to discard the data in phase two and estimate the OT treatment effect by only using data from phase one. The remaining data will be a double-blind randomized parallel design trial. We will estimate the OT treatment effect in comparison to the *P* (i.e., ${\tau }_{O}-{\tau }_{P}$) using the following simplified linear model (after removing the data in phase two).

$${y}_{i1}=\mu +{\tau }_{O}I\left({g}_{i1}=O\right)+{\tau }_{P}I\left({g}_{i1}=P\right)+{\sum }_{j=1}^{J}{\beta }_{j}{x}_{ij} +{\varepsilon }_{it}$$

A 50% reduction in self-reported pain intensity will demonstrate a clinically meaningful effect of OT compared to *P*.

### Methods in analysis to handle protocol non-adherence and any statistical methods to handle missing data {20c}

The randomized data (OT versus P) will be compared in missing patterns in the primary outcomes including reasons for missing data, timing of missing data, and distributions of baseline covariates and earlier outcomes. We will consider the following approaches to impute each of the primary outcomes within each phase respectively: (1) the last-observation-carried-forward method; (2) missing primary outcome predicted by a fitted regression model using demographic and baseline clinical variables; (3) missing primary outcome predicted by baseline and available follow-up outcomes on a fitted regression model. The imputation method for the primary intent-to-treat analysis will be selected using cross-validation performed on the participants with complete data. Specifically, we will evaluate imputation accuracy based on 2000 repetitions that randomly leave out 20% of the complete samples. In addition, to consider the uncertainty due to missing values, we will apply a multiple imputation procedure to generate multiple imputed data sets using the selected imputation approach, and combine results from analyses of these data sets, e.g., to provide mean and variance of the treatment effect estimates. No imputation is planned for secondary outcomes. Missing data in secondary outcomes will be considered missing. For a subject to achieve a given secondary endpoint, that endpoint must be observed.

### Plans to give access to the full protocol, participant-level data and statistical code {31c}

After trial completion and reporting of main study findings (i.e., specific aims), all data will be made publicly available upon reasonable request to the study principal investigators. Datasets and statistical code will also be publicly available after manuscripts are submitted for peer review.

### Oversight and monitoring

#### Composition of the coordinating center and trial steering committee {5d}

The UCOPE trial is a collaboration between laboratories in the UF Health Science Center (i.e. The Phenotyping & Assessment in Neuroscience- PAIN Lab directed by Dr. Cruz-Almeida) and the College of Liberal Arts & Sciences (i.e., The Socioemotional Aging Lab directed by Dr. Ebner). The PAIN lab provided organizational and day-to-day support for running the trial with guidance and oversight particularly in all aspects of oxytocin administration provided by Dr. Ebner’s lab. Frequent communication by study staff with the investigative team via weekly study meetings, individual lab meetings by Drs. Cruz-Almeida and Ebner, as well as daily emails, telephone calls, and text as needed.

### Composition of the data monitoring committee, its role and reporting structure {21a}

The UCOPE Study employs a two-member (i.e., a clinical pain researcher and a physician) Data and Safety Monitoring Board (DSMB), which reviews study progress and AEs to monitor the risk–benefit ratio of the trial. Twice per year, the DSMB receives a report that details progress with recruitment and retention, data completeness and quality, AEs, and any other reportable events. The study team meets with the DSMB to review the report, and the DSMB issues its findings and recommendations. DSMB members are independent from the sponsor and investigators and have no competing interests in the study.

### Adverse event reporting and harms {22}

We use a centralized system for safety monitoring, including tracking and reporting of adverse events (AEs). During each intervention period, participants complete a questionnaire weekly assessing symptoms that may represent AEs resulting from study procedures (Table 2). Study staff record information regarding AEs using a standardized form, which includes information regarding seriousness, expectedness, and relatedness of the AE. Reporting of AEs follows NIH and IRB guidelines.

**Table 2:** Adverse event symptom log of participants in the UCOPE study

Symptom	Yes or No	Severity Rating: Mild/Moderate/Severe	Increased or Decreased	Duration (in days): 1 to 7 days	Comments
Head					
Fever					
Drowsiness/Sleepiness					
Fainting					
Fatigue					
Light Headedness/Vertigo					
Headaches					
Seizures					
Other:					
Ear, Nose, Throat					
Sore Throat					
Dry Throat					
Hoarseness					
Nasal Irritation					
Runny Nose					
Nose Bleeds					
Sensitivity to Smells					
Stuffed-up Nose/Congestion					
Sneezing					
Upper Respiratory Infection					
Earache					
Tearing of the Eyes					
Other:					
Chest/Cardiovascular					
Heart Rate Changes/Palpitations					
Shortness of Breath					
Chest Pains					
Cough with Phlegm					
Changes in Blood Pressure					
Other:					
Digestive System					
Nausea/Vomiting					
Abdominal/Stomach Pain					
Changes in Urination Frequency					
Changes in Urine Color					
Constipation					
Diarrhea					
Change in Appetite					
Other:					
Skin					
Hives					
Skin Rash/Itching					
Skin Tingling					
Other:					
Emotional Responses and Motivation					
Feelings of Anger/Aggression					
Changes in Energy					
Changes in Attention/Focus					
Feelings of Stress or Anxiety					
Changes in Mood					
Changes in Sexual Drive/Performance					
Other:					
Other					
Leg Shaking					
Abnormal Sensation/Tingling of Limb					
Other:					
Females Only					
Abnormal Uterine Contractions/Cramping					
Spotting					
Other:					

### Frequency and plans for auditing trial conduct {23}

There were no planned procedures for auditing trial conduct, except as may be required by the UF IRB or the sponsor.

### Plans for communicating important protocol amendments to relevant parties (e.g. trial participants, ethical committees) {25}

Protocol modifications (e.g., changes to eligibility criteria, outcomes, analyses) to relevant parties (e.g., investigators, IRB, trial participants, trial registries, journals, regulators) will be communicated via email as well as changes in the myIRB system.

### Dissemination plans {31a}

Investigators will communicate trial results to participants, healthcare professionals, the public, and other relevant groups via peer-review publications.

## Discussion

OA represents the leading cause of pain and disability among older adults, and existing treatments fail to adequately reduce pain and improve function for a large proportion of OA sufferers. Our overarching working hypothesis is that older adults with chronic knee OA may benefit therapeutically from OT’s multiple pain-modulatory mechanisms. To test this account, the UCOPE Study was designed as a mechanistic, proof-of-principle, clinical trial to test the central hypothesis that a 4-week OT intervention is feasible and will be accepted by community-dwelling older adults. Further, we expect that OT administration will impact pain-related brain biochemistry and shift beneficially pain modulatory balance among older adults with symptomatic knee OA, producing greater OA-related pain relief. In pursing these goals, the UCOPE Study will provide novel experimental information regarding the mechanisms underlying OT responses to pain and its treatment, opening the door to future tailoring of pain treatments in older age.

### Trial status

Protocol Version 10 was IRB approved on 11/5/2020 3:19 PM, and recruitment began on October 2019. Recruitment was initially planned to be completed by summer 2024, but was actually completed on February 2024 with actual study follow-up ending on December 2024.

## Data Availability

All study investigators and regulatory bodies will have access to the final trial dataset. Any data required to support the protocol can be supplied on request.

## Abbreviations

AE: Adverse events
CTSI: Clinical and Translational Science Institute
DSMB: Data and Safety Monitoring Board
IRB: Institutional Review Board
OA: Osteoarthritis
OT: Oxytocin
QST: Quantitative sensory testing
UCOPE: Understanding Cognition, Oxytocin and Pain in Elders
UF: University of Florida
